# *Vibrio cholerae* genome isolated from the Nebraska salt marshes contains several antibiotic resistance markers

**DOI:** 10.1128/mra.00007-25

**Published:** 2025-03-27

**Authors:** John A. Kyndt

**Affiliations:** 1 College of Science and Technology, Bellevue University 52617, Bellevue, Nebraska, USA; University of Maryland School of Medicine, Baltimore, Maryland, USA

**Keywords:** *Vibrio cholerae*, salt marsh, antibiotic resistance, halophiles

## Abstract

A new strain of *Vibrio cholerae* was isolated from the Nebraska Salt Marshes and its genome sequenced. The genome shows several potential virulence factors and antibiotic resistance markers, which illustrates that multidrug-resistant pathogenic *V. cholerae* can be found in remote environments.

## ANNOUNCEMENT

*Vibrio cholerae* is an enteric pathogen that causes the acute watery diarrheal disease cholera. Several strains naturally live in water, and the world is currently in the midst of the seventh cholera pandemic (1). The World Health Organization has estimated that there are 1.3 to 4.0 million cases and 21,000 to 143,000 deaths from cholera worldwide each year, with cases rising in recent years (2, https://www.who.int/news-room/fact-sheets/detail/cholera). Some strains of *Vibrio* are becoming more resistant to many commonly used antibiotics (https://www.who.int/news-room/fact-sheets/detail/cholera), and a highly drug-resistant strain of cholera is now found in patients globally (3).

Microbial samples were collected from the Nebraska salt marshes near Lincoln, Nebraska (Lat. 40°52′54.42″N; Lon. 96°39′35.82″W), in October 2024, as part of an ongoing project that studies seasonal environmental microbiome fluctuations (4). Samples were collected in sterile 50 mL tubes from the edge of the Eastern Marsh Wren and stored at 4°C overnight. Then, 100 µL of the samples was used to inoculate RCV minimal media agar plates (5), supplemented with biotin (15 µg/L), nicotinic acid (1 mg/L), and NaCl (10 g/L), and grown aerobically at 35°C. After 2 days of aerobic growth, a brownish colony was picked and repeatedly transferred to obtain visibly pure cultures. A colony was used for genomic DNA extraction using the GeneJet DNA isolation kit (Thermo Scientific). DNA analysis using QuBit and NanoDrop showed a 260/280 absorption ratio of 1.81. The sequencing libraries were prepared using the Illumina DNA Library Prep kit and sequenced by an Illumina MiniSeq using 500 µL of a 1.8pM library. Paired-end (2 × 150 bp) sequencing generated 1,220,664 reads and 184 Mbp. Quality filtration (>Q30) and adapter trimming were performed by the MiniSeq software (v.2.2.1), and quality control of the reads was performed using FASTQC (version 1.0.0) within BaseSpace (Illumina), using a k-mer size of 5 and contamination filtering. The genome was assembled *de novo* through BV-BRC (6) using Unicycler (v0.4.8) (7). This resulted in a genome of 4,160,897 bp. The final genome was 100% complete and no contamination according to CheckM (v1.1.6) (8) and was annotated by NCBI PGAP (v6.6) (9). The genome characteristics are summarized in Table 1. Default parameters were used for all software applications unless otherwise noted.

**TABLE 1:** Overview of genome features of the genomes of *Vibrio* species related to *Vibrio cholerae* strain *NSM^a^*

Species	Size	% GC	Coverage	Contigs	N50	CDS	tRNAs	ANI %	Accession	Habitat	Ref
*Vibrio cholerae NSM*	4.2 Mb	47.4	46×	99	259,308	3,779	84	–	JBKFFD000000000	Salt marsh	This paper
*Vibrio cholerae ATCC 14035*	4.0 Mb	47.5	N/A	68	177,973	3,698	70	97.7	JHXR00000000	Human	(10)
*Vibrio metoecus YB5B04*	4.0 Mb	46.9	718×	50	291,368	3,785	67	86.4	LBGP01000000	Pond water	(11)
*Vibrio mimicus MB451*	4.3 Mb	46.4	8.5×	3	2,971,217	3,891	97	84.8	ADAF01000000	Human	(12)
*Vibrio fluvialis ATCC 33809*	4.8 Mb	49.9	493×	2	3,155,838	4,542	108	74.3	CP014034	Human	(13)
*Vibrio diabolicus SE1*	5.1 Mb	44.8	25×	41	694,617	4,815	62	71.7	JBCLWK010000000	Coastal lagoon	(14)

^*a*^ ANI percentage is based on bidirectional ANIb values to strain NSM, calculated using JSpecies (15).

Average percentage nucleotide identity analysis of the *V. cholerae* NSM genome compared with closely related genomes was performed using JSpecies (v4.1.1) (15). This showed strain NSM closest related to *Vibrio cholerae*, while other species were far below the arbitrary species cutoff (95%) (Table 1).

The BV-BRC comprehensive genome analysis identified over 100 virulence factors (with identity >90% and BLASP E-values < 2e−88) and 36 potential antibiotic resistance markers (with identity >81% and BLASP E-values < 1e−294), using the Victors/VFDB and PATRIC/CARD reference databases (6, 16–18). Of particular interest among the AMR targets is the tetracycline resistance MFS efflux pump Tet(35) (19) (>99% identity to tetracycline efflux genes from other *Vibrio* strains). Tetracyclines have long been the antibiotics of choice for treating severe cholera effectively worldwide. However, tetracycline-resistant strains of *V. cholerae* are being increasingly reported worldwide (20, 21). Although these resistant strains have been responsible for major epidemics in Latin America, Asia, and Africa, the widespread presence of tetracycline resistance in *Vibrio cholerae* is still understudied (22, 23), making the presence of multiple resistance genes in this new isolate from high saline and alkaline environments even more relevant.

## Data Availability

This Whole Genome Shotgun project has been deposited at DDBJ/ENA/GenBank under accession JBKFFD000000000. The version described in this paper is version JBKFFD010000000. The raw sequencing reads have been submitted to SRA and the corresponding accession number is SRR31807841.
